# Reward-Related Decision-Making in Current and Past Disordered Gambling: Implications for Impulsive Choice and Risk Preference in the Maintenance of Gambling Disorder

**DOI:** 10.3389/fnbeh.2021.758329

**Published:** 2021-10-29

**Authors:** Magdalen G. Schluter, David C. Hodgins

**Affiliations:** Addictive Behaviours Laboratory, Department of Psychology, University of Calgary, Calgary, AB, Canada

**Keywords:** gambling disorder (GD), impulsive choice, risky choice, delay discounting, probabilistic discounting, impulsivity

## Abstract

Impulsive reward-related decision-making (RRDM) is robustly associated with gambling disorder (GD), although its role in the development and perpetuation of GD is still being investigated. This project sought to examine the possible roles of impulsive and risky choice, two aspects of RRDM, in the perpetuation of GD. Additionally, the potential moderating role of comorbid substance misuse was considered. A total of 434 participants with symptoms of current GD and symptoms of concurrent substance use disorder (SUD; *n* = 105), current GD with past SUD (*n* = 98), past GD with current SUD (*n* = 53), or past GD with past substance use disorder (SUD; *n* = 92), and 96 healthy controls were recruited through MTurk. Participants completed a randomly adjusting delay discounting (a measure of impulsive choice) and probabilistic discounting (a measure of risky choice) task and self-report questionnaires of gambling participation, GD and SUD symptomology, and trait impulsivity. Although control participants showed significantly greater delay discounting compared to individuals with a current or history of GD, no significant group differences emerged between individuals with current GD or a history of GD. Individuals with current GD showed significantly less probabilistic discounting compared to individuals with a history of GD and control participants showed the greatest rates of probabilistic discounting. These effects remained after controlling for lifetime gambling symptom severity and trait impulsivity. Overall, these findings suggest a potential maintaining role of risky choice in gambling disorder, but do not support a maintaining role for impulsive choice.

## Introduction

Gambling shares many conceptual, neurobiological, and clinical similarities with substance use including associations with various facets of impulsivity (Grant et al., 2010). Although most individuals are social gamblers, gambling can lead to significant social, psychological, and financial harms. Gambling disorder (GD) is defined as “persistent and recurrent problematic gambling behavior leading to clinically significant impairment or distress,” indicated by symptoms including repeated unsuccessful efforts to cut back or stop gambling, preoccupation with gambling, and gambling with increasing amounts of money to achieve the same desired effect (American Psychiatric Association, 2013). The prevalence of GD is estimated to be 0.6% of the adult Canadian population (Williams et al., 2021) and is highly comorbid with substance misuse (SUDs; Petry et al., 2005). Given the harms that can result from GD, there is a clear need to understand both risk factors for developing and those involved in maintaining disordered gambling behavior.

Impulsive reward-related decision-making (RRDM) may be one such factor (Petry and Madden, 2010), although its precise role is still being investigated. RRDM involves cognitive and affective operations where the costs and benefits of potential rewards are weighed to, ideally, maximize potential benefits while minimizing the costs (Piantadosi et al., 2016). All individuals devalue larger, costly rewards to a certain extent, but this devaluation is particularly pronounced among individuals with addictive disorders (Alessi and Petry, 2003; Bickel et al., 2007). In fact, aberrations in RRDM are a hallmark of addiction (MacKillop et al., 2011) and is an increasingly popular area of research for understanding factors involved in GD. Two aspects of RRDM that may be particularly relevant for GD are delay and probabilistic discounting.

Delay discounting reflects the process by which future rewards are devalued as the delay before their receipt increases (Madden and Bickel, 2010). It provides a measure of impulsive choice, which is the relative preference for smaller, immediate rewards over larger-delayed rewards. It is one of the most studied types of RRDM, perhaps because life is rife with situations in which these types of decisions must be made. Consider the example from Madden and Johnson (2010): You have won a lawsuit, and you are guaranteed an annuity of $100,000 which cannot be cashed for 10 months. You can choose to sell this annuity and receive cash immediately, but you will receive less than the $100,000. A decision to take the smaller-sooner reward would indicate that the value of the large reward has been devalued, or its subjective value has been discounted from its nominal value (Green and Myerson, 2004; MacKillop et al., 2011). Impulsive choice is typically assessed using a discounting task in which the participant is asked to choose between smaller-sooner rewards and larger-later rewards. The value of the smaller reward at which you are indifferent about receiving it or opting for the larger reward (and its associated cost) reflects the subjective value of the larger reward. Indifference points are estimated for different temporal delays are plotted to yield a discounting curve and the rate at which the delayed reward is discounted (slope of subjective value per unit increase in delay) provides an index of impulsive choice. A steeper discounting curve reflects a greater relative preference for smaller-immediate rewards over the larger-later rewards, and greater impulsive choice.

Probabilistic discounting is a measure of risky choice and reflects how quickly the subjective value of a reward is discounted from its nominal value based on uncertainty of its receipt. Imagine that you have been offered employment at two different startup companies. One guarantees a salary of $100,000 per year, and the second offers company shares that could be worth significantly more than $100,000, but this is not certain. The choice to work for company shares would reflect a greater preference for the larger, uncertain monetary outcome, and greater risky choice. Risky choice may also be assessed with discounting tasks where the participant chooses between smaller-certain rewards and larger rewards of varying uncertainty. The rate at which the uncertain reward is discounted provides an index of risky choice, which is operationalized as a relative preference for large-uncertain rewards over small-certain rewards (Petry, 2012). A shallower discounting curve (i.e., less discounting) reflects a greater preference for the larger, uncertain reward, and greater risky choice.

Impulsive and risky choice are thought to underlie problematic gambling behavior. Relative to healthy controls, individuals with GD consistently demonstrate greater impulsive choice (Amlung et al., 2017) and risky choice (Kyonka and Schutte, 2018). Several meta-analytic studies have found moderate to large effect sizes for the association between GD and delay or probabilistic discounting. For example, MacKillop et al. (2011) reported a large effect size (*d* = 0.79) for the association between delay discounting rates and disordered gambling. The association between delay discounting rates and gambling severity remained among individuals without GD, though the size of the effect was attenuated (*d* = 0.41). Kyonka and Schutte (2018) also found a large effect size (Hedges’ g = 0.79) for the association between probabilistic discounting and disordered gambling. These studies highlight the robust association between impulsive and risky choice and GD. However, it is not yet clear how these facets of RRDM contribute to the development and/or maintenance of GD.

A substantial body of research suggests that impulsive choice may reflect both a risk factor for the development of addictive disorders and contribute to the maintenance of an addiction once it has developed. Much of this research has been done in animal models. For example, rats with a greater preference for immediate rewards are more likely to self-administer cocaine (Anker et al., 2009) as well as nicotine (Diergaarde et al., 2008) compared to rats with shallower DD. Although sparse, a similar pattern has been observed in human longitudinal research. Greater delay discounting in high school students predicts future smoking behavior (Audrain-McGovern et al., 2009), and greater delay discounting predicts future drug use among current drug users (MacKillop and Kahler, 2009). Significantly less delay discounting among abstinent drug users has been found compared to currently using drug users (Bretteville-Jensen, 1999; Kirby and Petry, 2004) and within the same individuals after treatment (Landes et al., 2012; Secades-Villa et al., 2014). However, little research has examined the etiological role of delay discounting in GD, and even less research has considered the role of probability discounting.

To our knowledge, only one study has investigated the possible roles of impulsive and risky choice simultaneously in the development and/or maintenance of GD. Petry (2012) evaluated delay and probabilistic discounting among a treatment-seeking sample of individuals with GD prior to beginning treatment. Baseline probabilistic discounting rates, but not delay discounting rates were related to abstinence in the short term and at 12-months post-treatment. Specifically, less PD discounting (i.e., greater risky choice) at baseline was associated with greater likelihood of maintaining abstinence. Petry (2012) proposed that those participants with higher risky choice may have had a greater awareness of their problems with gambling, and thus may have preferred abstinence goals over controlled gambling. While this study is the first, and only, to look at discounting and treatment outcomes in GD, the sample was composed of treatment-seeking individuals with severe ratings of gambling behaviors. Whether these findings generalize to the larger group of individuals with GD who do not seek treatment is unclear (Slutske, 2006). Additionally, this study did not include post-treatment discounting assessments. Several studies have found rates of delay discounting improve with continued abstinence in SUDs (Landes et al., 2012; Secades-Villa et al., 2014). As such, baseline rates alone may not predict who achieves long-term abstinence. If impulsive and risky choice decrease over treatment in individuals most likely to achieve long-term abstinence, then individuals who have maintained long-term abstinence from gambling should show less delay discounting (lower impulsive choice) and greater probabilistic discounting (lower risky choice) than individuals who currently have a GD.

Few studies have also considered the presence of concurrent substance misuse in currently abstinent individuals with GD. The importance of considering concurrent substance misuse is particularly salient for several reasons. First, GD is highly comorbid with SUDs (25–73%; Petry, 2005; Lorains et al., 2011). Second, individuals with GD and concurrent SUDs show much greater delay discounting compared to non-substance-misusing individuals with GD (Petry, 2001). Given that addictive substances and problem gambling may contribute to dysfunction in the same reward circuitry in the brain, one possibility is that they have additive or synergistic effects, resulting in greater impulsive choice among individuals with concurrent addictions. Thus, individuals who misuse substances may continue to show greater delay discounting, even among individuals who have stopped gambling. However, substance misuse has not been found to impact probabilistic discounting rates (Andrade and Petry, 2012). For example, Konova et al. (2020) found that risk tolerance, as assessed by a probability discounting task, did not predict future opioid use among individuals with an opioid use disorder. Given that delay and probabilistic discounting are associated with different patterns of activation in brain reward circuitry (Miedl et al., 2012), it is possible that substance-related modulation of mesolimbic dopamine transmission differentially impacts the circuits involved in impulsive choice. Reward-related learning also modulates dopamine transmission, and it is possible that learning associated with probabilistic discounting is more relevant in the context of GD, as individuals who gamble are constantly exposed to scenarios that involve making choices about possible rewards in the face of varying outcomes (Wiehler and Peters, 2015).

In sum, delay and probabilistic discounting are two aspects of RRDM that are robustly associated with GD. However, RRDM is multi-faceted, and the cost and time required for longitudinal research creates a barrier to delineating the contributions of various aspects of RRDM to the development and perpetuation of GD. This project sought to investigate the associations between two aspects of RRDM, delay and probabilistic discounting, among individuals who screened positive for a likely history of GD but who were currently abstinent and among those who currently screened positive for a likely GD. The purpose of this comparison was to consider their possible roles in the development or maintenance of GD and to inform future longitudinal research. Theoretically, if impulsive and risky choice confer only a risk to the development of GD but not its maintenance, then whether an individual has achieved long-term abstinence should have no impact on measures of delay and probabilistic discounting. However, if impulsive and risky choice are involved in perpetuating problematic gambling once an individual has developed GD, then we would expect to see greater delay and less probabilistic discounting among individuals with a likely current GD, compared to those who have achieved long-term cessation of gambling.

We also sought to consider the potential interacting contribution of likely current or past SUD on the relationship between GD and delay discounting rates. In light of the research findings discussed above the following predictions were made: (1) individuals with (likely) current GD would show the greatest rates of impulsive choice as measured on a delay discounting task compared with individuals with a (likely) past GD and controls; (2) (likely) SUD status would moderate the association between GD status and impulsive choice such individuals with a current SUD but past GD would continue to show greater impulsive choice than individuals who had recovered from both GD and SUD.; (3) individuals with a current GD would show greater risky choice on a probability discounting task compared to individuals with a history of GD and controls; and (4) we would not see a significant interaction between GD and SUD status on risky choice.

## Materials and Methods

### Sample

Participants were composed of adults from Amazon’s Mechanical Turk worker pool. MTurk is a popular crowdsourcing platform that is increasingly used for psychological research (Shapiro et al., 2013). Consistent with recommendations for recruiting specific samples through MTurk (Chandler and Shapiro, 2016), a two-stage recruitment procedure was utilized. Participation was limited to individuals who resided in the United States and had an approval rating of at least 95%.

*A priori* power analysis indicated that a sample of ∼100 participants per group is required to detect a modest effect size (*f* = 0.14; from Amlung et al. (2017) at *p* = 0.05 with a minimum power of 0.80. To account for failed validity checks on the discounting task, we aimed to recruit 10 to 15% additional participants, leading to a goal of 625 participants. In part 1, 3,539 individuals participated. Of these participants, 1,649 were eligible to complete part 2, and 683 did so (Supplementary Figure 1). These participants comprised 586 participants in four target groups, and 97 healthy controls with no history of substance misuse or problematic gambling. Target groups consisted of the following: (1) current GD and current SUD, (2) current GD and past SUD, (3) past GD and current SUD, or (4) past GD and current SUD.

### Recruitment Procedures

Like procedures previously employed and recommended for recruiting clinical samples on MTurk (Chandler and Shapiro, 2016; Schluter et al., 2018). In part 1, participants completed questionnaires about recent gambling behaviors, current and lifetime GD symptoms, and current and lifetime SUD symptoms. Participants who met eligibility criteria for one of the five groups and passed the first validity check were invited to complete part 2. In part 2, participants completed additional validity items, the Delay Discounting Task, and a measure of impulsive personality traits. Part 2 was hosted through Inquisit, a platform for precision measuring in behavioral paradigms that can be integrated with MTurk and administered over the web. MTurk workers were compensated approximately $0.10 per minute for their participation.

#### Eligibility Criteria

To be eligible to complete part 2, participants must (a) have demonstrated consistent responding, (b) fall in to one of the five groups, and (c) be over the age of 21. Due to difficulty in distinguishing problematic from non-problematic substance use/gambling among individuals without a likely current addictive disorder, individuals with a past addictive disorder were only eligible if they had not engaged in the addictive behavior in the past 12 months.

Participants who reported at least four symptoms consistent with DSM-5 criteria for GD in the past 12-months were classified as individuals with likely *current GD*. Those who reported at least four symptoms in their lifetime but no gambling in the past 12-months were classified as experiencing a likely *past GD*. Participants who reported at least two symptoms consistent with DSM-5 criteria for SUDs were classified as individuals experiencing likely *current SUD*, and those who reported at least two symptoms in their lifetime but had not used the substance in the past 12-months, were classified as individuals with likely *past SUD.* Individuals with no self-reported symptoms of problematic gambling or substance misuse, and no substance use more than five times (except for alcohol) in any 12-month period in the past were classified as *controls*.

To address concerns with symptom misreporting in MTurk samples (Chandler and Paolacci, 2017), NODS-CLiP responses were compared to responses to identical questions presented later in the survey as part of the NODS questionnaire. The purpose of this was to detect individuals who anticipated the nature of these screening items and misreported symptoms to continue the study. Participants who responded inconsistently were ineligible for part 2. Among individuals who completed part 2, the NODS-CLiP was presented once more. Participants who endorsed a symptom in part 1 but not in part 2 were excluded from analysis.

### Measures

#### NODS-CLiP

Participants first completed the NODS-CLiP (Toce-Gerstein et al., 2009), a rapid screen for disordered and problem gambling in adults. The three questions, derived from the NODS (see below) pertain to loss of control, lying, and preoccupation with gambling. Participants who endorsed at least one item went on to complete the remaining questionnaires. Control participants must not have endorsed any symptoms.

#### Demographic Questionnaire

A lab-developed questionnaire assessed demographic information including age, gender, marital status, level of education, and household income.

#### Gambling Participation Instrument (GPI)

The GPI (Williams et al., 2017) is a self-report measure of gambling involvement in the past 12-months across a variety of gambling behaviors: lottery or raffle tickets, instant lottery tickets or games, electronic gambling machines, casino table games, sports betting, speculative financial market activities, and other types of gambling. Participants are asked to report the frequency with which they participated in specific gambling activities, the amount of time spent in a typical month, and amount of money spent in a typical month.

#### National Opinion Research Center DSM-IV Screen for Gambling Problems (NODS)

The NODS (Gerstein et al., 1999) is a self-report screening measure of both current and lifetime problem gambling based on DSM-IV criteria. Seventeen questions for lifetime and 17 corresponding past-year items are scored yes or no and measure 10 GD criteria. A total score is calculated based on the number of criteria the person meets. Consistent with DSM-5 diagnostic criteria for GD, the criterion of engagement in illegal activity was removed from analysis, and a threshold of 4 was used to indicate likely GD. This approach shows excellent sensitivity and specificity (>97%) and hit rates (>88%) across a variety of samples (Petry et al., 2013).

#### UPPS-P Impulsive Behavior Scale

The UPPS-P (Lynam et al., 2006) is a 59-item self-report measure of personality traits that lead to impulsive behaviors. It assesses 5 subscales; negative urgency reflects the tendency to act rashly in response to strong negative emotions; (lack of) premeditation reflects the ability to think through possible consequences before acting; (lack of) perseverance reflects the ability to persist in completing tasks; sensation seeking reflects the preference for excitement and stimulation; finally, positive urgency reflects the tendency to act rashly in response to strong positive emotions.

#### Substance Use Disorder

Current and lifetime SUD was assessed using questions derived from the World Health Organization (WHO) Composite International Diagnostic Interview Version 2.1 (CIDI; World Health Organization, 1998). The CIDI is a diagnostic interview for ICD-10 and DSM-IV disorders that can be computer self-administered (Sunderland et al., 2011). Separate modules assess tobacco, alcohol, and eight other classes of substances.

Due to time constraints, participants were asked which class of substances they felt was currently or previously the most problematic for them and only completed questions for that class of substances. Following these questions, a final question asked if they felt they were currently experiencing similar problems with another substance, or at some point in the past.

#### Discounting Task

Impulsive and risky choice were measured in part 2 using a modified version of the Richards et al. (1999) delay and probabilistic discounting task. Participants are asked to make choices between smaller-immediate and larger-later rewards, and between smaller-certain and larger-uncertain rewards. The Richards task has high test-retest reliability (*r* = 0.89; Weafer et al., 2013) and similar results when potentially real or hypothetical rewards are used, supporting its ecological validity (Richards et al., 1999).

To be consistent with other paradigms using hypothetical rewards and to provide suitable variability in possible scores, the standard delayed reward was set at $1000, and seven temporal delays were presented: 1 day, 1 week, 1 month, 3 months, 1 year, 5 years, and 25 years (Koffarnus and Bickel, 2014). The five probabilities were as follows: 100, 90, 75, 50, and 25%. The magnitude of the rewards was systematically varied using an adjustment algorithm to estimate indifference points, where the immediate/certain reward is equal to the subjective value of the delayed/uncertain reward (Myerson et al., 2001). The magnitude of the rewards was systematically varied using an adjustment algorithm to estimate indifference points, where the immediate/certain reward is equal to the subjective value of the delayed/uncertain reward (Myerson et al., 2001). The delay and probability trials were presented with a mixed design; For a given trial, participants could be presented with either a delay or probability. For each delay or probability, participants completed trials until an indifference point was determined, or until 30 trials were administered.

### Data Analysis

All analyses were conducted in R (R Core Team, 2013). Baseline characteristics between participants were summarized using means, standard deviations, counts, and percentages. These were compared for eligible participants who participated or did not participate in part 2, as well across the five groups in the final sample using the Fisher exact test for categorical variables or one-way ANOVA with the *car* package (Fox et al., 2012) for continuous variables. Regression analyses were conducted to test for between-group differences in delay and probabilistic discounting among the four target groups and control group. Demographic variables (age, education, marital status, gender, and household income) and trait impulsivity (UPPS-P) were included in the models as covariates. Models including only GD status were first run. Next, SUD status and an interaction term between GD and SUD status were added. All linear models were developed and tested using the *lm()* and *Anova ()* functions of the *car* package. Graphs were produced using the *ggplot2* package.

The area under the empirical discounting curve (AUC) was used as the discounting parameter and calculated using the *pracma* package (Borchers, 2019) where smaller values indicate steeper discounting. The AUC model is recommended over other mathematical models (e.g., exponential decay or hyperbolic discounting) when looking for between-group differences for several reasons: AUC frequency distributions are approximately normally distributed (Myerson et al., 2001), AUC does not require a fitted regression curve, and the field currently lacks concensus regarding the exact mathematical function underlying discounting (Borges et al., 2016). For delay discounting, steeper (i.e., greater) discounting reflected in smaller a AUC_*d*_ indicates greater impulsive choice. For probabilistic discounting, shallower (i.e., less) discounting resulting in a larger AUC_*p*_ reflects greater risky choice. Visual inspection indicated that AUC_*d*_ values in the current study were not normally distributed. Therefore, a base-10 logarithmic transformation was applied to the delay values. The original AUC was retained as the probabilistic discounting parameter estimate, as the AUC values were approximately evenly distributed.

To ensure logically consistent responding in the discounting task, two criteria were applied. First, the indifferent point at the first delay must be at least 100 greater than the indifference point at the longest delay. Second, no more than one indifference point could be 200 greater than the point preceding it. These criteria are widely used in the delay discounting literature (Johnson and Bickel, 2008), including in our own work using clinical, crowdsourced samples (Schluter et al., 2018).

## Results

### Preliminary Analysis

A total of 3,539 individuals participated in part 1 of the study. Of these participants, 1,649 were eligible to participate in part 2. Of those excluded, 1002 participants (53.01%) showed inconsistency in their endorsement of GD symptoms. Much of the inconsistent responding was characterized by endorsement of items on the NODS-CLiP that were not endorsed later in the NODS. Other reasons for ineligibility included not meeting eligibility criteria for both current or past GD and SUD or controls (*n* = 871), or not providing their worker ID to be recontacted (*n* = 17; Supplementary Figure 1).

Of the 1,649 participants who were invited to complete part 2, 681 did so. Individuals who completed part 2 had a mean age 1.25 years older than those who did not complete part 2, *t*(3470) = 2.84, *p* = 0.004, but did not differ in demographics otherwise (*p*s < 0.12). One-hundred twenty-three participants who completed part 2 showed inconsistently endorsed GD symptoms, characterized by negative endorsement of a symptom on the NODS-CLiP that had been endorsed in part 1, and 66 participants were excluded for logically inconsistent responding on the discounting task. Fifty-eight participants who met diagnostic criteria for a past SUD reported feeling that they were currently experiencing similar problems with another substance. These participants were also excluded from analysis, leading to 434 participants in the final sample.

Demographic characteristics of the final sample by target group are included in Table 1. The groups were compared on demographic data using the Fisher exact test (gender, education, ethnicity, marital status, and employment) or one-way analysis of variance (age). Except for marital status and employment (*p*s < 0.02) the five target groups did not differ significantly. On average, 10 out of 12 indifference points were estimated for each participant for the discounting task. The groups did not differ significantly in the average number of indifference points estimated, *F*(1,432) = 2.02, *p* = 0.15, suggesting that the points were missing at random. Where possible, missing indifference points were replaced with the average value of the indifference point before and after the missing value. In the case where two indifference points were missing, this was not possible.

**TABLE 1:** Demographic information for participants across groups.

	Current GD and current SUD (*n* = 105)	Past GD and current SUD (*n* = 98)	Current GD and past SUD (*n* = 53)	Past GD and past SUD (*n* = 82)	Control participants (*n* = 96)
Age – M (*SD*)	36.11 (9.13)	36.38 (8.19)	38.86 (11.08)	36.59 (10.80)	38.81 (12.09)
**Gender – N (%)**					
Male	48 (45.71)	35	25	40	56
Female	55 (52.38)	60	26	39	39
Other/Prefer not to disclose	2 (1.90)	3	2	3	1
**Income – N (%)**					
Under $10,000	1	6	4	4	3
$10,000 to $39,000	38	30	18	35	25
$40,000 to $69,000	36	35	19	23	32
$70,000 to $99,000	30	25	12	19	36
Unknown	0	2	0	1	0
**Education – N (%)**					
High school diploma or less	18	29	16	21	17
Trades or apprenticeship	4	5	3	2	2
College	18	12	7	13	7
University below bachelors	12	13	4	9	10
Bachelors	46	27	15	25	40
Graduate degree	7	10	8	12	20
Unknown	0	2	0	0	0
**Ethnicity – N (%)**					
Caucasian	79	80	49	68	81
Chinese	0	2	0	2	2
South Asian	1	3	0	2	0
Black	14	10	3	11	6
Fillipino/Pacific Islander	1	2	0	1	1
Latin American	12	2	2	7	6
Southeast Asian	0	1	0	0	1
Japanese	2	0	0	1	2
Korean	1	0	0	0	0
Aboriginal	0	2	1	0	1
Other	1	0	0	1	0
**Employment – N (%)**					
Employed:*					
*Full-time*	83	72	39	53	53
*Part-time*	11	11	6	11	17
Unemployed	4	10	7	10	7
Student	4	5	1	4	9
Retired	2	0	1	2	2
Other*	2	2	0	3	10
Marital status – N (%)*					
Unmarried	41	41	25	33	38
Legally married	39	36	14	34	50
Common-law	13	6	2	5	1
Separated	5	4	3	3	0
Divorced	7	9	9	7	7
Unknown	0	2	0	0	0

GD, gambling disorder; SUD, substance use disorder. *p < 0.05.

The discounting parameters were compared to other measures of gambling activity, symptom counts, and trait impulsivity with Pearson or Spearman correlation coefficients (Table 2). Across the whole sample, measures of delay and probabilistic discounting showed small-to-moderate correlations with lifetime gambling symptom severity (NODS; *r*s = −0.2 and 0.17). and past-year symptom severity (*r*s = −0.11 and 0.2). Lifetime substance misuse severity was also correlated with delay (*r* = −0.17) and probabilistic discounting (*r* = 0.14), and past-year substance misuse severity showed a small association with delay discounting (*r* = −0.11). Excluding control participants, probabilistic discounting continued to show a significant correlation with past-year gambling symptom severity (*r* = 0.16). Delay discounting was also correlated with lifetime gambling symptom severity (*r* = −0.1), but other correlations were attenuated.

**TABLE 2:** Correlations between diagnostic symptom severity, trait impulsivity, measures of gambling activity, delay discounting, and probabilistic discounting.

	1	2	3	4	5	6	7	8	9	10	11	12
1. AUC_*log*_	−	0.04	**−0.2**	**−0.11**	**−0.17**	**−0.11**	**−0.17**	**−0.15**	**−0.11**	**−0.06**	**−0.12**	**−0.16**
2. AUC_*p*_	**0.11**	−	**0.17**	0.20	**0.14**	0.08	**0.16**	**0.16**	0.05	**0.19**	**0.21**	**0.21**
3. NODS–Lifetime	−**0.10**	< 0.01	−	**0.45**	**0.85**	**0.46**	**0.54**	**0.41**	**0.15**	**0.31**	**0.51**	**0.51**
4. NODS–Past year	–0.06	**0.16**	**0.25**	−	**0.37**	**0.29**	**0.30**	**0.31**	**0.11**	**0.19**	**0.32**	**0.32**
5. CIDI–Lifetime	–0.02	–0.04	**0.23**	0.07	−	**0.43**	**0.54**	**0.41**	**0.18**	**0.34**	**0.50**	**0.52**
6. CIDI–Past year	–0.04	< 0.01	**0.19**	**0.15**	**0.14**	−	**0.36**	**0.26**	**0.15**	**0.21**	**0.35**	**0.36**
7. NURG	–0.01	0.05	**0.22**	**0.14**	**0.22**	**0.21**	−	**0.69**	**0.49**	**0.43**	**0.82**	**0.89**
8. PREM	–0.05	**0.10**	**0.12**	**0.20**	**0.15**	**0.12**	**0.62**	−	**0.53**	**0.53**	**0.70**	**0.85**
9. PERS	–0.05	0.02	< 0.01	0.06	0.08	**0.10**	**0.46**	**0.51**	−	**0.11**	**0.41**	**0.58**
10. SS	< 0.01	**0.17**	**0.14**	**0.10**	**0.20**	**0.11**	**0.38**	**0.49**	0.09	−	**0.50**	**0.68**
11. PURG	0.01	**0.16**	**0.23**	**0.19**	**0.22**	**0.21**	**0.78**	**0.64**	**0.36**	**0.46**	−	**0.91**
12. UPPS-P Total	–0.02	**0.14**	**0.20**	**0.18**	**0.24**	**0.21**	**0.85**	**0.83**	**0.56**	**0.66**	**0.89**	−

*Values in the upper triangle calculated from entire sample. Values in the lower triangle calculated from target group participants only. NODS, National Opinion Research Center DSM-IV Screen for Gambling Problems; CIDI, World Health Organization Composite International Diagnostic Interview substance misuse symptoms; AUC_d_, area under the delay discounting curve; AUC_log_, log transformed AUC_d_; AUC_p_, area under the probabilistic discounting curve. Values in bold are significant at p = 0.05.*

Across the entire sample, both delay and probabilistic discounting showed significant associations with the UPPS-P total score (*r*s = −0.16 and 0.21), and the following subscales: negative urgency (*r*s = −0.17 and 0.16), positive urgency (*r*s = −0.12 and 0.21), and lack of premeditation (*r*s = −0.15 and 0.16). Sensation seeking was also significantly associated with probabilistic discounting (*r* = 0.19), and lack of perseverance was correlated with delay discounting (*r* = −0.11). On measures of gambling activity, delay discounting showed no significant associations in either the entire sample or when excluding controls. However, probabilistic discounting was significantly associated with the number of gambling types engaged in, money spent, and the amount of time spent gambling in both the entire sample (*r*s = 0.14–0.17) and excluding controls (*r*s = 0.11–0.18).

### Delay Discounting

Across the entire sample, AUC_*d*_ ranged from 0.04 to 0.91 (*M* = 0.15; *SD* = 0.17). Log transformed AUC values ranged from −2.71 to −0.04 (*M* = −1.06, *SD* = 0.47). Table 3 reports the descriptive statistics for the raw an transformed AUC values across groups. Controlling for demographic variables (age, gender, marital status, education, and household income) and trait impulsivity (UPPS-P total score), AUC_*log*_ values showed a significant difference across groups based on GD status, *F*(2,368) = 3.35, *p* = 0.04, partial η^2^ = 0.02.

**TABLE 3:** Descriptive statistics of the raw and transformed area under the delay discounting curve.

	Raw AUC			Log-transformed AUC		
Group	*M*	*SD*	MED	*M*	*SD*	MED
Current GD and current SUD	0.13	0.17	0.07	–1.14	0.45	–1.14
Past GD and current SUD	0.16	0.19	0.08	–1.07	0.52	–1.07
Current GD and past SUD	0.16	0.19	0.09	–1.04	0.44	–1.07
Past GD and past SUD	0.11	0.13	0.08	–1.13	0.40	–1.08
Controls	0.19	0.19	0.11	–0.92	0.46	–0.94

*GD, gambling disorder; SUD, substance use disorder.*

In support of hypothesis 1, individuals with a current GD (*M* = −0.1.11, *SD* = 0.45) showed significantly greater delay discounting compared to control participants (*M* = −0.92, *SD* = 0.46), est = 0.18 (*SE* = 0.03), 95% CI [0.04,0.33], and *p* = 0.01. However, individuals with a current GD did not show significantly greater delay discounting overall when compared to individuals with a history of GD (*M* = −1.10, *SD* = 0.47), *p* = 0.67; Individuals with a history of GD continued to show significantly greater delay discounting overall compared to control participants, est = 0.16 (*SE* = 0.07), 95% CI [0.02,0.29], and *p* = 0.02.

Overall between-group differences were observed only at delays of 3 months and 1 year. Specifically, at a delay of 3 months, individuals with a current GD showed a smaller indifference point (*M* = 533.54, *SD* = 391.57) than both control participants (*M* = 758.54, *SD* = 304.33), est = 161.95 (SE = 58.82), 95% CI [46.28, 277.63], *p* = 0.02, and individuals with a history of GD (*M* = 636.89, *SD* = 365.04) est = 91.61 (SE = 43.08), 95% CI [8.90, 178.33], *p* = 0.03. However, individuals with a history of GD did not demonstrate a significantly different indifference point than control participants, *p* = 0.21. at 1 year, only individuals with a current GD showed a smaller indifference point (*M* = 315.00, *SD* = 336.48), than controls (*M* = 499.58, *SD* = 362.60), est = 133.78 (*SE* = 55.28), 95% CI [25.07, 242.49], *p* = 0.02.

The linear model showed issues with aliased coefficients when SUD status was included in the model, suggesting significant shared variance. As such, the model with SUD status could be compared to the model without SUD using the *anova()* function but could not be examined directly. Comparison of the two models suggested that the more complex model did not more adequately capture the data than the model with only DG status included. *F*(1,370) = 0.61, *p* = 0.44. As such, hypothesis 2 was not supported. Additionally, when lifetime GD symptom severity was added to the model without SUD status, the effect of GD status on AUC_*log*_ was no longer significant, *F*(2,367) = 0.06, *p* = 0.94, partial η^2^ < 0.001.

#### Supplemental Analyses

Given the disproportionate contributions of longer delays to the AUC, Borges and colleagues recently (2016) proposed a modified version of the AUC where the base-10 logarithmic transformation is applied to the delay values themselves, which are then normalized. In a supplemental analysis we estimated the delay discounting parameter with the modified AUC method. The results were like those reported in the core analyses above. One difference we noted was that the difference between controls and participants with a past GD trended towards significance (*p* = −0.054), though remained statistically insignificant.

To further consider whether GD and SUD are distinct when it comes to delay discounting, an additional supplemental analysis was run which predicted variance in delay discounting from SUD status only. Controlling for demographic variables (age, gender, marital status, education and household income) and trait impulsivity (UPPS-P total score), AUC_*log*_ values showed a significant difference across groups based on SUD status, *F*(2,371) = 3.54, *p* = 0.03, partial η^2^ = 0.02. Control participants (*M* = −0.92, *SD* = −0.46) showed significantly greater delay discounting than individuals with a likely SUD (*M* = −1.18, *SD* = 0.49), est = −0.19 (*SE* = 0.07), 95% CI [−0.33, −0.05], *p* = 0.008, or with a likely past SUD (*M* = −1.10, *SD* = 0.42), est = −0.14 (*SE* = 0.07), 95% CI [0.0.28, −0.001], *p* = 0.05. However, individuals with a current SUD did not differ significantly from individuals with a past SUD, est = 0.05 (*SE* = 0.06), 95% CI [−0.06,0.16], *p* = 0.39.

### Probabilistic Discounting

AUC_*p*_ ranged from 0.05 to 0.84 across the entire sample (*M* = 0.48, *SD* = 0.17). Table 4 reports the descriptive statistics for the AUC_*p*_ across GD groups. Controlling for demographic variables (age, gender, marital status, education, and household income) and trait impulsivity (UPPS-P total score), GD status predicted probabilistic discounting, *F*(2,364) = 7.01, *p* = 0.001, partial η^2^ = 0.04. Consistent with hypothesis 3, individuals with current GD showed less probabilistic discounting (*M* = 0.52, *SD* = 0.17) compared to control participants (*M* = 0.25, *SD* = 0.15), est = 0.09 (SE = 0.03), 95% CI [0.00,0.14], *p* < 0.001 and compared to individuals with a history of GD (*M* = 0.47, *SD* = 0.17), est = 0.06, 95% CI [0.00,0.10], *p* = 0.003. These effects remained after also controlling for the severity of lifetime GD symptoms, *F*(2,364) = 6.69, *p* = 0.001, partial η^2^ = 0.04. Consistent with hypothesis 4, the addition of SUD status to the model did not more adequately capture the data than the model with only GD status included, *F*(1,363) = 0.88, *p* = 0.35.

**TABLE 4:** Descriptive statistics of the area under the probabilistic discounting curve.

Group	*M*	*SD*	MED
Current GD	0.52	0.17	0.50
Past GD	0.47	0.17	0.47
Controls	0.42	0.15	0.38

*GD, gambling disorder.*

## Discussion

Impulsive and risky choice are critical aspects of RRDM (Myerson et al., 2003) that are thought to be involved in GD. Impulsive choice is also thought to be involved in the etiology and maintenance of substance misuse (MacKillop et al., 2011). However, their roles in the context of GD are still being investigated. This project investigated the associations between impulsive and risky choice and GD status (current vs. past vs. controls) as well as the potential moderating role of substance misuse status (current vs. past).

Consistent with our first hypothesis, individuals with a current GD showed greater delay discounting curves indicating greater impulsive choice than control participants. However, individuals with a history of GD also showed greater delay discounting compared to control participants, suggesting that even individuals who have achieved long-term cessation of gambling may continue to show a preference for more immediate rewards over larger, delayed rewards. Additionally, when lifetime symptom severity of GD was controlled for, the effect of GD status on delay discounting was no longer significant, suggesting that between group differences reflects greater gambling severity rather than GD status. Additionally, only at 3-months and 1-year did we see significant between-group differences based on GD status. We theorized that if impulsive choice is involved in the maintenance of problem gambling behavior, then we would expect to see a difference in delay discounting between individuals currently experiencing a problem and those who had successfully recovered. In contrast, if impulsive choice is not critical to the maintenance, then whether someone has achieved long-term abstinence should have little impact on discounting rates. While indifference points were significantly different at 3-months and 1-year, the overall dates of discounting did not differ significantly at shorter or longer delays. As such, our results suggest that impulsive choice generally may have greater relevance to the development of GD than in perpetuating a problem once it has developed, as rates of delay discounting were not significantly different among people with either a current or past GD. This result is counter to previous research with smokers, which found that individuals who had successfully quit showed similar rates of delay discounting compared to individuals who had never smoked (Bickel et al., 1999). The discrepancy between these studies is somewhat surprising, as researchers have suggested that impulsive choice may be a component of transdiagnostic impulsivity (Amlung et al., 2017). Our results suggest that impulsive choice may be less amenable to change among individuals with GD than the literature has suggested for substance misuse. Although substances and gambling are associated with dysfunction in reward pathways in the brain, substances may lead to reversible changes in these pathways, whereas gambling is not associated with such drug-induced changes.

We also predicted that substance misuse status would moderate the effect of GD status on rates of delay discounting, but this was not observed. Addition of substance misuse to the model did not improve the amount of variance in delay discounting rates already captured by GD status. Lifetime and past-year symptom severity of substance misuse was significantly correlated with delay discounting rates (*r*s = −0.17 and −0.11), and the strength of the association was like those for lifetime and past-year gambling severity (*r*s = −0.20 and −0.11). However, we could not make any conclusions regarding the moderating role of substance misuse.

Consistent with our third hypothesis, individuals with current GD showed significantly greater risky choice, reflected in less probabilistic discounting, compared to individuals with a history of GD and compared to controls. While individuals with past GD showed greater risky choice than control participants, they discounted probabilistic rewards more greatly than individuals with current GD, suggesting that these individuals were more risk averse. These effects remained after controlling for severity of lifetime GD symptoms, suggesting that our findings were not simply a function of greater problem gambling severity. Based on these findings, it is possible that risky choice plays a role in the perpetuation of problematic gambling behavior once a disorder has developed. If this were not the case, then we would have expected to see similar rates of discounting in individuals who had developed a GD, regardless of whether they had recovered.

There are several possible explanations for why individuals with past GD showed greater risk aversion compared to individuals with a current GD; they could have experienced lower risk preference prior to stopping gambling, or became more risk averse as they recovered from GD. This second possibility is more consistent with the results of the previous study by Petry (2012), which found that greater risk preference predicted better abstinence outcomes following treatment for GD. If individuals who achieve long-term abstinence have greater awareness of their gambling behaviors when they decide to stop gambling than individuals who continue to gamble, then their decision-making may be more amenable to change. Thus, these individuals develop greater risk aversion, whereas individuals who continue to gamble maintain their preference for risky choices. Of note, this sample consisted of individuals who self-reported that they had a problem with gambling currently or in the past, as did the sample recruited in Petry (2012), and these findings may not generalize to individuals who do not recognize their gambling as a problem.

Overall, the present findings highlight the role that risky choice, measured by a probabilistic discounting task, may play in the perpetuation of gambling-related problems. Risky choice may therefore reflect an important intervention target. Interestingly, individuals with GD appear to have accurate understanding of probabilities (Ligneul et al., 2013) so education about odds and probabilities may not improve gambling outcomes. Rather, these risk preferences may be strongly affect-driven (Mukherjee, 2010). Supporting this explanation, Shead et al. (2008) found that individuals who expect gambling to enhance positive mood tend to make riskier choices. Additionally, probabilistic discounting was significantly associated with positive urgency in the present study, which reflects the tendency to engage in risky behavior when experiencing intense positive emotion (Lynam et al., 2006). It also showed positive associations with other facets of affective-motivational impulsivity that we have previously identified to potentially contribute to greater levels of gambling severity, as well as more impulsive RRDM. Thus, risky choice may be a response to intense emotion, and interventions that target the fusion of these emotional experiences to risk preferences may be more beneficial than education about odds and probabilities. It is important to note that risky choice, as measured by probabilistic discounting, assesses one sub-process of risky decision making Other tasks, such as the Iowa Gambling Task (IGT; Bechara et al., 1994) assess related, but distinct facets. Interestingly, individuals with SUD show impairments on the IGT, though not greater risky choice on probability discounting. One possibility for this is that tasks such as the IGT require integration of general risk preference with learning and strategic components of decision-making behavior (Bechara et al., 1994; Brevers et al., 2013).

This study has several limitations. First, MTurk workers are a non-representative sample. They tend to be young, well-educated, and less religious than the overall population (Paolacci and Chandler, 2014). Second, self-report surveys carry issues of inattention and malingering. The extant literature provides some confidence for the reliability and validity of self-report data on MTurk. Data collected through MTurk has demonstrated high convergent and concurrent validity (Chandler and Shapiro, 2016), scale reliability (Buhrmester et al., 2011), test-retest reliability (Chandler and Shapiro, 2016; Kim and Hodgins, 2017), and comparable effect sizes to those seen in the existing literature on a variety of psychological measures (Shapiro et al., 2013). Many participants were excluded for inconsistent responding in part 1, which also highlights problems with symptom misreporting in MTurk samples. Fortunately, the NODS-CLiP in parts 1 and 2 allowed many participants to be identified, mitigating the risk that individuals in the target groups were composed of individuals feigning symptoms of GD. Third, although we are using questionnaires that are aligned with diagnostic criteria, we do not have a “gold standard” for addiction diagnosis. Fourth, violation of the logical consistency criteria may be related to many factors, not just inattention (Rung et al., 2018). We excluded all participants that violated these criteria which may have excluded some participants who were responding attentively. However, adoption of this criteria strengthens our confidence that the final sample of participants was comprised of attentive individuals. Fifth, treatment history was not explicitly assessed and, as such, we cannot draw any conclusions regarding the relationship between risky and impulsive choice, and treatment history. Given that our results suggest that risky choice contributes to the maintenance of GD, it is possible that individuals with greater risky choice undergo more change attempts before ultimately being successful with stopping gambling. Future research may benefit from a careful consideration of treatment seeking history among individuals with ongoing gambling problems and investigating the association with probabilistic discounting rates. Finally, because the study is not longitudinal, we cannot know whether individuals with past GD merely have lower risk preference to begin with, or whether risk preference changes over treatment/with continued abstinence. Future research should investigate these possibilities.

## Data Availability Statement

The datasets presented in this article are not readily available because participants did not consent to have their raw data made available. Requests to access the datasets should be directed to MS, magdalen.schluter@ucalgary.ca.

## Ethics Statement

The studies involving human participants were reviewed and approved by Conjoint Faculties Research Ethics Board (CFREB) at the University of Calgary. The patients/participants provided their written informed consent to participate in this study.

## Author Contributions

MS wrote the first draft of the manuscript. DH wrote parts of the manuscript and edited subsequent versions. Both authors contributed to the article and approved the submitted version.

## Conflict of Interest

The authors declare that the research was conducted in the absence of any commercial or financial relationships that could be construed as a potential conflict of interest.

## Publisher’s Note

All claims expressed in this article are solely those of the authors and do not necessarily represent those of their affiliated organizations, or those of the publisher, the editors and the reviewers. Any product that may be evaluated in this article, or claim that may be made by its manufacturer, is not guaranteed or endorsed by the publisher.
