# Supplementary material for: Reward-Related Decision-Making in Current and Past Disordered Gambling: Implications for Impulsive Choice and Risk Preference in the Maintenance of Gambling Disorder
Source: Front Behav Neurosci. 2021 Oct 29;15:758329. doi: 10.3389/fnbeh.2021.758329 (PMC8586647; doi:10.3389/fnbeh.2021.758329)
Supplement: Supplementary file 1 [file Data_Sheet_1.docx]

Analyzed (n=434)

Excluded (n=247)

Inconsistent responding (n=123)

Logically inconsistent responding (n=66)

Past SUD, but reported current problems (n=58)

Did not complete part 2 (n=968)

Completed part 2

(n=681)

Excluded (n = 1,890)

Did not respond consistently (n=1002)

Responded consistently but did not meet eligibility criteria (n=871)

Worker ID not provided (n=17)

Invited to complete part 2

(n=1,649)

Completed Part 1

(n=3,539)

**Supplementary Figure 1.** Flow chart of participants excluded at each stage of screening.

**Regression Analysis Formulas in R Software**

**Delay Discounting**

**linear model formula with GD included**

lm1<- lm(log10(area)~gender + age + maritalstatus + education + household_income + UPPSPtotal + DG_Status, data=x1)

**linear model adding gambling severity to the model**

lm2<- lm(log10(area)~gender + age + maritalstatus + education + household_income + UPPSPtotal + DG_Status + NODS_lifetime, data=x1)

**additional model with SUD status included instead of GD status**

lm3<- lm(log10(area)~gender + age + maritalstatus + education + household_income + UPPSPtotal + SU_Status, data=x1)

**Probabilistic Discounting**

**model with GD included**

lm1<- lm(probarea~gender + age + maritalstatus + education + household_income + UPPSPtotal + DG_Status, data=x1)

**model with added gambling severity**

lm2<- lm(probarea~gender + age + maritalstatus + education + household_income + UPPSPtotal + DG_Status + NODS_Lifetime, data=x1)
